# Validity and reliability of the Malay version of the Structured Migraine Interview (SMI) Questionnaire

**DOI:** 10.1186/s10194-015-0509-5

**Published:** 2015-03-14

**Authors:** Munvar Miya Shaik, Norul Badriah Hassan, Huay Lin Tan, Shalini Bhaskar, Siew Hua Gan

**Affiliations:** Human Genome Centre, School of Medical Sciences, Universiti Sains Malaysia, 16150 Kubang Kerian, Kelantan Malaysia; Department of Pharmacology, School of Medical Sciences, Universiti Sains Malaysia, 16150 Kubang Kerian, Kelantan Malaysia; Gleneagles Medical Centre, No 1, Jalan Pangkor, 10050 Penang, Malaysia

**Keywords:** Malay, SMI, Migraine, Questionnaire, Validation

## Abstract

**Background:**

The Structured Migraine Interview (SMI) is a valid and reliable instrument for migraine diagnosis. However, a Malay version of the SMI is not available to be applied to the local Malaysian population. This study was designed to access the validity and reliability of a new Malay version of the SMI questionnaire.

**Methods:**

Patients with headache attending the Neurology Clinic, Hospital Universiti Sains Malaysia, Kelantan, Malaysia, were screened against the inclusion/exclusion criteria before recruitment. A standard translation procedure was used to translate and adapt the questionnaire into the Malay language. The translated version was tested for face, content and construct validities. Subsequently, validity and reliability studies were conducted (1^st^ compilation), followed by retesting seven days later (2^nd^ compilation).

**Results:**

A total of 157 patients between 15 and 60 years of age were enrolled in this study. The kappa value was 0.70 (*p* < 0.001) with high sensitivity (0.97) and specificity (0.63). The misclassification rate was 0.15, with a positive predictive value of 0.82 and a negative predictive value of 0.92. The positive likelihood ratio was 2.62, while the negative likelihood ratio was 0.05. The Cronbach’s alpha was 0.93 (1^st^ compilation) and 0.90 (2^nd^ compilation), respectively. The Spearman’s correlation coefficient ranged from 0.86 (Question 4) to 0.95 (Question 1). The overall concordance for item 1 was very high (97%), followed by item 4 (83%), item 2 (71%) and finally item 3 (64%).

**Conclusion:**

The Malay version of the SMI questionnaire is comparable to the English version in terms of validity and reliability. It was highly reliable with good internal consistency and can be used for the diagnosis of migraine in clinical settings in Malaysia.

## Background

Migraine is a common, painful and disabling condition characterized by recurrent, unilateral and pulsatile attacks of headaches of moderate or severe intensity [1]. Most migraine attacks begin at puberty and affect those between 35 and 45 years of age. The prevalence of migraine among women is higher (5% to 25%) when compared with men (only 2% to 10%) [2,3]. The prevalence peaks in the middle age but was reported to be lower in adolescents and among the elderly (older than 60 years of age) [3].

According to the International Headache Society (IHS) diagnostic criteria, migraine can be divided into two subtypes: migraine with aura (MA) and migraine without aura (MO), with the latter being the more common type (80%) [3]. MA presents with reversible symptoms such as blurred vision, vertigo or hallucinations that precede or accompany the headache. This situation is different from MO in which such symptoms are absent [4]. Nearly 31% of migraine populations are reported to have attack frequencies of three or more per month, with 54% reporting severe impairment [3]. Disability due to recurrent headache is a major concern in migraine patients because it impairs quality of life (QOL) and work capacity [5]. The World Health Organization reported that migraine is ranked as the 19^th^ cause of years living with disability [6].

Surprisingly, nearly 60% of migraine sufferers are unaware that they have migraine [7]. Furthermore, the diagnosis of migraine tends to be limited to a smaller population due to the lack of proper healthcare, diagnostic tools and neurologists. These situations may contribute to the delay in providing treatment and may affect migraine patients’ QOL and duration of quality working hours. Accordingly, migraine may become a burden to the patient and to the patient’s family members, as well as to the country, either directly or indirectly.

A migraine diagnosis is important because it reduces the burden of symptoms on patients and improves the prognosis for migraine and its associated psychiatric co-morbidities. However, migraine screening among the general population is seldom conducted due to lack of awareness and proper tools. Because there are no known biological markers identified for the objective assessment of migraine, migraine diagnosis currently relies solely on careful history documentation and on the absence of significant signs upon physical examination.

The IHS published diagnostic criteria for all types of headache disorders including migraine [8], which can assist clinicians to diagnose migraine in a systematic manner. However, subjective diagnosis may lead to inaccuracies, as diagnoses may vary from one clinician to another. Therefore, to avoid inconsistency and lack of agreement between clinicians, a structured and reliable method to define clinical symptoms and phenotypes such as depression and headache is very important to improve diagnostic accuracy.

Several questionnaires are available for the diagnoses of migraine including ID-Migraine Screener (IDMS) [9], deCODE Migraine Questionnaire (DMQ3) [10], the Dutch headache questionnaire [11] and the German headache questionnaire [12]. The most popular questionnaire (IDMS) has only three items, but it fails to identify the aura symptoms. In addition, this questionnaire is limited only to the clinical diagnosis and may not be useful for genetic research [9]. The DMQ3 questionnaire can be useful in diagnosing both MA and MO. However, it contains 56 items, which can be tedious for the patients to answer [10]. In addition, the Dutch and German headache questionnaires are not preferred because of their poor sensitivity among patients [11,12]. In comparison, the Structured Migraine Interview (SMI) Questionnaire [13] is a simple self-administered questionnaire that can diagnose migraine according to the guidelines of the IHS and was therefore selected for this study.

The SMI questionnaire was designed to determine whether a patient has suffered from migraine at any time in his/her life [13]. The questions were designed to include individual items from the International Classification of Headache Disorders (ICHD) criteria for migraine [4]. This instrument consisted of two sections; the first consisted of four items for migraine diagnosis, and the second contained six items for screening of symptoms associated with migraine. The diagnosis of MA is based on having the diagnostic features of MO and must include at least five migraine attacks in one’s lifetime. In addition, for the diagnosis of MA, there must be at least two attacks accompanied by aura symptoms as determined by question 4.

To date, there is no Malay version of SMI for use in the local setting. For accurate and reliable responses and for greater relevance to the local setting, a questionnaire in the National Malay language is required for the diagnosis of migraine. Therefore, this study aimed to develop and validate a new Malay-translated version of the SMI to be applied to the local population of migraine sufferers.

## Methods

**Figure 1:**
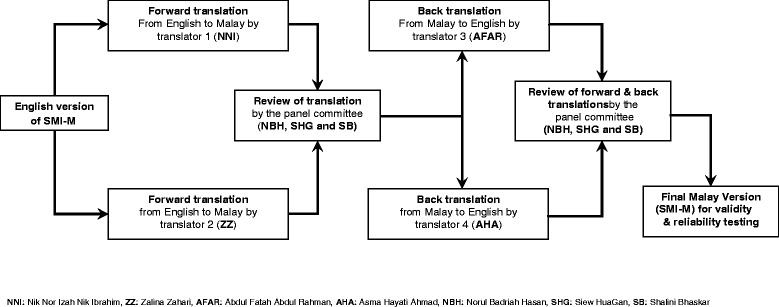
**Forward and back translations of the SMI-M Questionnaire.**

The translation of the original English SMI version into the Malay version was conducted according to a standardized protocol [14] (Figure 1). Only the first part of the original SMI was translated and validated in this study.

### Forward and back translations

Two independent translators (NNI and ZZ) performed the forward translations. Both translators are native Malay speakers with a good command of English who speak multiple dialects of the Malay language and are bicultural. The forward translated versions were evaluated by a panel of experts (NBH, SHG and SB) to further verify the conceptual, idiomatic, semantic, and cross-cultural equivalence to the English version.

The final forward translation was then back-translated into the English language by two different independent back-translators (AFAR and AHA). Both translators were unaware of the concepts and purpose of the questionnaire or the nature of the study. These translators were bicultural, native Malay speakers with multiple dialects and with good command of the English language. A special panel (NBH and SHG) reviewed the forward- and back-translated versions to produce the final draft of the Malay version of the SMI-M.

### Face and content validity of the SMI-M

To determine face validity, the SMI-M was administered to 30 patients who have headache and are aged between 15 and 60 years. This step is essential to ensure the quality of the questionnaire and also to acquire proper feedback from patients when answering the translated questions, especially in understanding the medical terminology used. During this period, no difficulties in understanding the questions were noted. Overall, the SMI-M questionnaire was easily understood and well accepted by both the patients and the experts alike. The content validity was then established by administering the SMI-M questionnaire to an expert (SB) in the Neurology Department for further evaluation. A special panel (NHB, SHG and SB) further reviewed, discussed and modified the questionnaire based on the noted problems to construct the final SMI-M questionnaire.

### Data collection

Patients with headache (n = 200) who registered with the Neurology Clinic of Hospital Universiti Sains Malaysia (HUSM) between January and December 2013 were randomly screened according to the inclusion and exclusion criteria. The inclusion criteria included patients ageing between 15 and 60 years who have been diagnosed with migraine based on the criteria of the *2*^*nd*^*edition* of the ICHD (ICHD-II), 2004 [1] or who are suspected to be suffering from headache. Patients with cardiovascular disorders, neurological disorders, head injuries, ophthalmological problems, menstrual migraine or who were pregnant were excluded. The study was approved by the Universiti Sains Malaysia Research and Ethical committee (ethical no: USMKK/PPP/JEPeM [231.3.(08)]) and complies with the Declaration of Helsinki.

**Figure 2:**
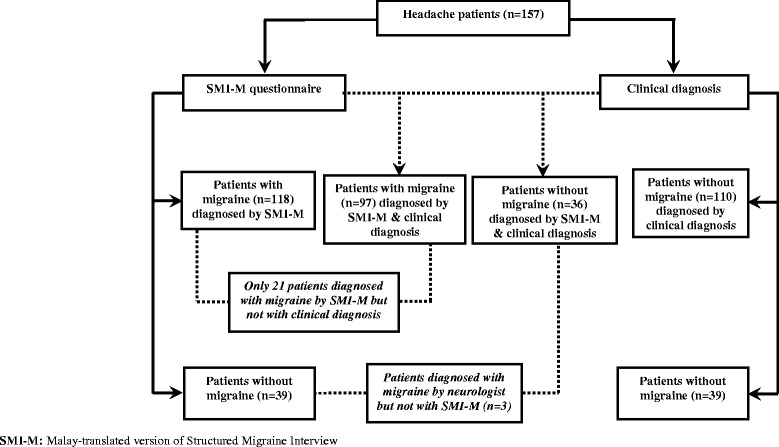
**Flowchart of data collection.**

After the initial screening process, 157 patients having headache agreed to participate in the study (Figure 2). They were verbally informed about the purpose of the study before being asked to complete the written informed consent forms, basic socio-demographic information form and SMI-M questionnaire. Following this step, all patients were evaluated by a headache specialist during their initial visits. Overall, patients were asked to complete the SMI-M twice: the first time during their visits to the clinic (1^st^ compilation) and the second time at home (2^nd^ compilation) where the completed SMI-M questionnaires were mailed back to researchers in standard stamped envelopes seven days following the completion of the 1^st^ compilation. Patients were only aware of their clinical diagnosis but not the diagnosis by SMI-M. In addition, patients were also asked to continue their regular medication if prescribed by the clinician.

### Statistical analysis

Kaiser-Meyer-Olkin (KMO) and the Barlett’s tests of sphericity were applied to estimate the sampling adequacy [15]. Sensitivity, specificity, misclassification rate, positive predictive value, negative predictive value, positive likelihood ratio and negative likelihood ratio of migraine diagnosis by SMI-M were further measured to test the validity of the SMI-M.

The internal consistency of the SMI-M score was assessed using Cronbach’s alpha (α). An α value of 0.7 is considered to be “acceptable”, while an α value of 0.8 or more indicates excellent internal consistency [16]. A test-retest reliability to measure consistency across the duration (i.e., between the 1^st^ and 2^nd^ compilations) of both total and individual question scores was evaluated using Spearman correlations. A Spearman correlation is conservative, as it is not usually influenced by outliers. The percentage of concordance of the 2^nd^ compilation compared with the 1^st^ was taken as the stability of the SMI-M. Statistical analysis was conducted using SPSS version 20.0 software (New York, USA).

## Results

### Validity and reliability of the SMI-M

A total of 157 patients with headache completed the administered questionnaires relatively quickly. None of the patients had any difficulties understanding or answering any parts of the SMI-M. The mean age of the patients was 26.81 (8.33) years and the number of years of education was 14.25 (2.81) (Table 1). In this study, the majority of the patients were students (56.7%) and singletons (70.7%). The majority (63.7%) of household incomes were average (between RM 801- RM 3000). Only female patients were recruited for the study, and all of them were of the Malay race, which is the dominant ethnic group in the Kelantan state of Malaysia. The sample was adequate as indicated by a KMO value of 0.85 and a significant Barlett’s test of sphericity (p < 0.001).

**Table 1:** **Demographicdata of the subjects**

	**n (157)**	**Percentage (%)**	**Mean**	**SD**
Age (years)			26.81	8.33
Education (years)			14.25	2.81
Relationship				
Single	111	70.7		
Married	43	27.4		
Divorced/Widowed	3	1.9		
Employment position				
Student	89	56.7		
Housewife	14	8.9		
Government job	40	25.5		
Private job	7	4.4		
Self-employed	5	3.2		
None	2	1.3		
Income (MYR)				
Below 450	5	3.2		
450-800	11	7.0		
801-1500	35	22.3		
1501-3000	65	41.4		
3001-6000	32	20.4		
Above 6000	9	5.7		

**MYR:** Malaysian ringgit.

**Figure 3:**
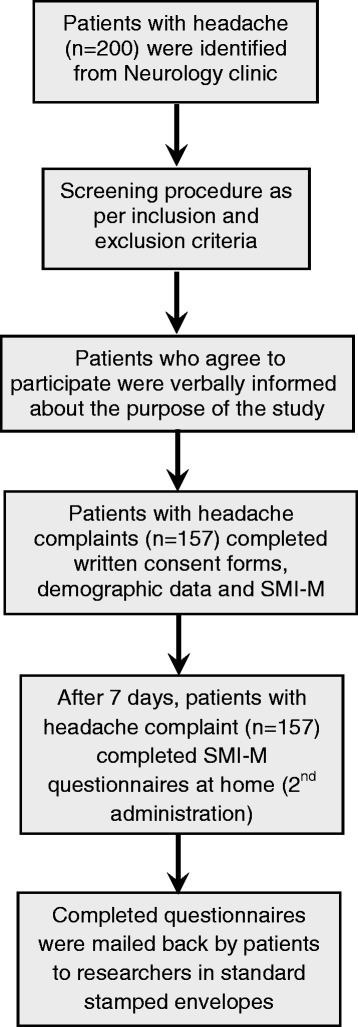
**Comparison of SMI-M and clinical diagnosis.**

Clinically, 63.7% of the patients were diagnosed as having migraine by the neurologist (SB) in contrast to 75.2%diagnosed as having migraine using the SMI-M questionnaire (Figure 3). In total, 22.9% of the patients were diagnosed as not having migraine by both the neurologist and the SMI-M questionnaire. However, 13.4% of the patients were diagnosed as having migraine using the SMI-M, and these same patients were diagnosed as not having migraine by the neurologist. The SMI-M failed to accurately diagnose three (1.9%) patients as having migraine as diagnosed by the neurologist. Therefore, the misclassification rate was 0.15, the positive predictive value was 0.82, the negative predictive value was 0.92, the positive likelihood ratio was 2.62, and the negative likelihood ratio was 0.05. In addition, the sensitivity was 0.97 and specificity was 0.63. The Kappa statistic value was 0.7 (*p* < 0.001) and was statistically significant (Table 2).

**Table 2:** **The SMI-M versus neurologist’s clinical diagnoses “gold standard”**

**SMI-M diagnoses**	**Clinical diagnoses**		**Total**
	**Migraine**	**No migraine**	
Migraine	97 (a)	21 (b)	118
No migraine	3 (c)	36 (d)	39
Total	100	57	157

**Note: Sensitivity:** a/(a + c) = 97 / (97 + 3) = 0.97.

**Specificity:** d/(b + d) = 36 / (36 = 15) = 0.63.

**Misclassification rate:** (b + c) / (a + b + c + d) = (21 + 3)/(97 + 21 + 3 + 36) = 0.15.

**Positive predictive value:** a/(a + b) = 97/(97 + 21) = 0.82.

**Negative predictive value:** d/(c + d) = 36 /(36 + 3) =0.92.

**Positive likelihood ratio:** Sensitivity / (1-specificity) = 0.97 / (1-0.63) = 2.62.

**Negative likelihood ratio:** (1-Sensitivity) / specificity = (1-0.97) / 0.63 = 0.048.

The Cronbach α values at the 1^st^ (0.93) and 2^nd^ compilations (0.90, *p* < 0.001) exceeded the 0.9 level, indicating excellent internal consistency. The Spearman’s correlations for the 1^st^ and 2^nd^ compilations were compared and ranged from 0.86 (for question 4) to 0.95 (for question 1) (Table 3). The overall concordance was calculated as the percentage of the 1^st^ and 2^nd^ compilations for each item (Table 4). The overall concordance for item 1 was very high (97%), followed by item 4 (83%), item 2 (71%) and item 3 (64%). For item 2 (moderate to severe headache accompanied by nausea and/or vomiting), approximately 85.71% of the patients changed their responses from the fourth option (Yes, 10+ times) to other options. For item 3 (moderate to severe headache accompanied by hypersensitivity to sound or light), approximately73.91% of the patients changed their responses from the fourth option (Yes, 10+ times) to other options.

**Table 3:** **Spearman correlation coefficient between test and retest of the SMI-M Questionnaire**

**Question**	**Spearman’s correlation coefficient**	***p*** **value**
1. Have you ever had recurrent headaches? [Pernahkah anda mengalami sakit kepala yang berulang-ulang?]	0.95	**<0.001**
2. Have you ever had moderate to severe headache accompanied by nausea and/or vomiting? [Pernahkah anda mengalami sakit kepala yang sederhana hingga teruk yang disertai rasa loya dan/atau muntah?]	0.87	**<0.001**
3. Have you ever had moderate to severe headache accompanied by hypersensitivity to sound or light? [Pernahkah anda mengalami sakit kepala yang sederhana hingga teruk yang disertai dengan hipersensitiviti kepada bunyi atau cahaya?]	0.88	**<0.001**
4. Have you ever had visual disturbances e.g., (Flashing lights, zigzag lines, blurred vision) Lasting 5-60 minutes followed by headache? [Pernahkah anda mengalami gangguan penglihatan contohnya (cahaya yang berkelip-kelip, garisan yang berbentuk zigzag, penglihatan yang kabur) berlanjutan selama 5 hingga 60 minit dan diikuti dengan sakit kepala?]	0.86	**<0.001**

*Correlation was significant at 0.05 level.

**SMI-M:** Malay-translated version of Structured Migraine Interview.

**Table 4:** **Changes of the SMI-M items response from the 1** ^**st**^ **and 2** ^**nd**^ **compilations**

***Q1. Have you ever had recurrent headaches?***						
	**Number and percentage of patients for each response at first compilation**	**Number and percentage of patients for each response at second compilation**				**Number and percentage of patients who changed grades between administrations**
		**No**		**Yes**		
**No**	32 (20.4%)	32 (100.0%)		0 (0.0%)		0 (0.0%)
**Yes**	125 (79.6%)	3 (2.4%)		122 (97.6%)		3 (100%)
**Total**	157 (100.0%)	35 (22.3%)		122 (77.7%)		3 (1.9%)
***Q2. Have you ever had moderate to severe headache accompanied by nausea and/or vomiting?***						
	**Number and percentage of patients for each response at first compilation**	**Number and percentage of patients for each response at second compilation**				**Number and percentage of patients who changed grades between administrations**
		**No, Never**	**Yes, 1-4 times**	**Yes, 5-9 times**	**Yes, 10+ times**	
**No, Never**	32 (20.4%)	31 (96.9%)	1 (3.1%)	0 (0.0%)	0 (0.0%)	1 (3.4%)
**Yes, 1-4 times**	94 (59.9%)	3 (3.2%)	82 (87.2%)	8 (8.5%)	1 (1.1%)	12 (41.4%)
**Yes, 5-9 times**	17 (10.8%)	0 (0.0%)	4 (23.5%)	13 (76.5%)	0 (0.0%)	4 (13.8%)
**Yes, 10+ times**	14 (8.9%)	0 (0.0%)	1 (7.1%)	11 (78.6%)	2 (14.3%)	12 (41.4%)
**Total**	157 (100.0%)	34 (21.7%)	88 (56.1%)	32 (20.4%)	3 (1.9%)	29 (18.5%)
***Q2. Have you ever had moderate to severe headache accompanied by hypersensitivity to sound or light?***						
	**Number and percentage of patients for each response at first compilation**	**Number and percentage of patients for each response at second compilation**				**Number and percentage of patients who changed grades between administrations**
		**No, Never**	**Yes, 1-4 times**	**Yes, 5-9 times**	**Yes, 10+ times**	
**No, Never**	45 (28.7%)	38 (84.4%)	7 (15.6%)	0 (0.0%)	0 (0.0%)	7 (19.4%)
**Yes, 1-4 times**	72 (45.9%)	3 (4.2%)	64 (88.9%)	5 (6.9%)	0 (0.0%)	8 (22.2%)
**Yes, 5-9 times**	17 (10.8%)	0 (0.0%)	3 (17.6%)	13 (76.5%)	1 (5.9%)	4 (11.1%)
**Yes, 10+ times**	23 (14.6%)	0 (0.0%)	2 (8.7%)	15 (65.2%)	6 (26.1%)	17 (47.2%)
**Total**	157 (100.0%)	41 (26.1%)	76 (48.4%)	33 (21.0%)	7 (4.5%)	36 (22.9%)
***Q4. Have you ever had visual disturbances e.g., (flashing lights, zigzag lines, blurred vision) lasting5-60 minutes followed by headache?***						
	**Number and percentage of patients for each response at first compilation**	**Number and percentage of patients for each response at second compilation**				**Number and percentage of patients who changed grades between administrations**
		**No, Never**	**Yes, once**		**Yes, 2+ times**	
**No, Never**	52 (33.1%)	49 (94.2%)	1 (1.9%)		2 (3.8%)	3 (17.7%)
**Yes, once**	51 (32.5%)	4 (7.8%)	46 (90.2%)		1 (2.0%)	5 (29.4%)
**Yes, 2+ times**	54 (34.4%)	2 (3.7%)	9 (16.7%)		43 (79.6%)	9 (52.9%)
**Total**	157 (100.0%)	41 (26.1%)	76 (48.4%)		33 (21.0%)	17 (10.8%)

**SMI-M:** Malay-translated version of Structured Migraine Interview.

## Discussion

In this study, we successfully developed and validated the SMI-M questionnaire, which is suitable to be applied to the local population. The present study indicated that the translated version of the SMI-M questionnaire was valid, reliable and could easily be applied for migraine diagnosis. It is also easy to understood and well accepted by patients with headache. The factor analysis with a good KMO value and a significant Barlett’s test of sphericity, indicated that there was an adequate sample size for the validation of the SMI-M questionnaire. The patients recruitment, study design and validation of SMI-M were similar with the original study in order to eliminate the bias when comparing the findings of both studies. To our knowledge, our study is the first migraine diagnostic study conducted in the South East Asia region.

### Validation of SMI-M questionnaire

Patients attending a neurology clinic are usually afflicted with severe headache associated with impairment so debilitating that a specialist’s intervention is required. During the 1^st^ compilation, patients were asked to report any problems related to the questionnaire. However, no difficulties in understanding and answering the SMI-M questionnaire were reported. In addition, no ongoing therapies (symptomatic or prophylactic) undertaken by the patients were altered during the course of the test-retest study to ensure that similar conditions were maintained for the patients in both compilations.

Migraine remains a clinical diagnosis based on the IHS’ migraine diagnosis criteria, which is considered to be the “gold standard”. Therefore, the findings from the SMI-M were compared with migraine diagnosis by the headache specialist in order to test the validity of SMI-M. The validity testing compared with the clinical diagnosis indicated that the SMI-M is a highly sensitive (0.97) instrument but was only moderately specific (0.63) in identifying migraine cases. Nevertheless, the sensitivity and specificity results of the SMI-M from this study were consistent with the original questionnaire.

The moderate value of kappa statistic (0.7) indicates a substantial agreement between SMI-M and the clinical diagnosis [17]. The kappa value in this study was slightly lower (0.70) than that in the original study (0.82). Previous reports suggested that regional differences may lead to variations in the clinical diagnosis. The difference in the kappa values between the original SMI and SMI-M may be due to differences in clinical practices between clinicians in the different regions [18]. Furthermore, variations between personal, organizational and systemic levels may also lead to variations in clinical practice [19]. The positive predictive value (0.82) was slightly lower than that of the original study (0.97). However, the positive predictive value depends upon the prevalence of the outcome of interest, which varies from one population to another [20]. Nevertheless, the negative predictive value in this study (0.92) was much higher than that of the original study (0.26), indicating that the SMI-M questionnaire rarely misclassified a migraine patient as a non-migraine patient [20].

The lower misclassification rate (0.15) indicates that SMI-M is not capable of labelling non-migraine patients as having migraine. A higher positive likelihood ratio of SMI-M (2.62) in this study when compared with the original SMI (2.07) suggested that there was a higher probability for a migraine patient to be correctly diagnosed using this questionnaire. A lower negative likelihood ratio of SMI-M (0.05) than the original SMI (0.22) makes SMI-M optimal in ruling out the chances of wrongly diagnosing migraine. Therefore, the SMI-M can help clinicians to decide whether a patient indeed has migraine [21].

The SMI-M has been reported to be comparable with the IDMS [9] and DMQ3 [10] but is better than the Dutch [11] and German headache questionnaires [12], which was also found in our study. For example, the sensitivity of SMI-M (0.97) was similar to that of the DMQ3 (0.99) but was higher than those of the IDMS (0.81), Dutch (0.51) and German headache questionnaires (0.63). In comparison with SMI-M, the DMQ3 contained 56 items, which can be tedious for patients to answer. In contrast, the SMI-M is very easy for patients to score when compared with the DMQ3, as it has only four items focusing specifically on migraine diagnosis. However, the IDMS questionnaire only has three items but does not contain any questions related to aura symptoms. Therefore, the SMI-M is a valid tool to be used in research for the identification of migraine in large samples where clinical interviews are less practical. In addition, the SMI-M should be applied to the local clinical settings for reconfirming the status of migraine. Nevertheless, some variations (whenever they exist) may be contributed by the study design and possibly also in the degree of strictness with which the ICHD-II criteria were applied in clinical the diagnosis.

### Reliability of SMI-M questionnaire

The internal consistency of the SMI-M questionnaire was excellent for both compilations. The higher value of Cronbach’s alpha indicated a higher degree to which all items in the SMI-M questionnaire measured single uni dimensional latent constructs. This value also indicated good questionnaire reliability in diagnosing migraine among Malay patients with headache. Although the patients were aware of their neurological diagnosis, the high correlation values based on Spearman’s statistical analysis indicated excellent test-retest reliability seen with the SMI-M questionnaire. In this study, comparing the reliability of the SMI-M with the original SMI questionnaire could not be conducted, as reliability studies were not reported in the original study.

Among all of the items, item 1 yielded the highest Spearman’s correlation, which may be attributed to the simplicity of the question pertaining to the existence of recurrent headache. However, item 4 yielded the lowest Spearman’s correlation coefficient, even though the correlation coefficient was excellent. This findings may be attributed to the 7 days gap, which may have allowed some time for the development of migraine-related events that may have affected some of the responses including MA symptoms in between the two compilations.

Nearly 36% of the patients changed their response choice from the 1^st^ to the 2^nd^ compilation, which again may be attributed to the 7 day gap between the compilations. It was observed that the change of symptoms or a new migraine attack could occur in a short period, which may be sustained for long hours and may contribute to the change in scores in a short time period especially among severe migraine patients. For item 1, only 3% of the patients changed their responses, but for item 3, 36% patients changed their responses between both compilations. The duration between both compilations may have allowed precipitations or changes in the symptoms, especially if patients are hypersensitive to light and sounds, which are common stimulants for migraine as investigated in question 3. Nevertheless, gaps between 2 days and two weeks have previously been reported to be ideal for test-retest reliability studies [22].

The second part of the original SMI questionnaire that was related to symptoms was not included in the SMI-M as it is simpler, less time consuming, and focuses only on diagnosis. Moreover, removing the second part of the questionnaire did not affect its validity or reliability.

### Limitations of the study

This sample was limited only to patients from the Malay race because there was no report of migraine patients from other races (Chinese, Indian and Siamese). All patients were females because no male patients with headache visited the clinic during the study period. Although care was taken with the translation and cross-cultural adaptation process to keep the originality, simplicity and clarity of the questionnaire, cross-cultural equivalence of the questionnaire is subjective and is difficult to measure precisely. Therefore, expert judgment was necessary to investigate whether the original and translated versions are equal.

## Conclusions

In conclusion, the SMI-M questionnaire maintains the conciseness and validity parameters of the English version, but is simpler and less time consuming. The reliability values of SMI-M were also exceptionally good, with a high concordance percentage between the 1^st^ and 2^nd^ administrations of the SMI-M. Therefore, the SMI-M is a good and useful self-assessment tool for theearly diagnosis of individuals with migraine.

## Abbreviations

DMQ3: DeCODE Migraine Questionnaire
ICHD: International classification of headache disorders
IDMS: ID-migraine screener
IHS: International headache society
KMO: Kaiser-Meyer Olkin
MA: Migraine with aura
MO: Migraine without aura
SMI: Structured migraine interview
MI: The Malay translated version of SMIQOL, quality of life
